# Research progress on AMPK in the pathogenesis and treatment of MASLD

**DOI:** 10.3389/fimmu.2025.1558041

**Published:** 2025-03-11

**Authors:** Jiang Feng, Li MengHuan, Yao TingTing, Yi XueJie, Gao HaiNing

**Affiliations:** ^1^ School of Exercise and Health, Shenyang Sport University, Shenyang, China; ^2^ School of Physical Education, Liaoning Normal University, Dalian, China

**Keywords:** NAFLD, MASLD, AMPK, lipid metabolism, inflammatory response

## Abstract

Metabolic dysfunction-associated steatotic liver disease (MASLD; formerly known as non-alcoholic fatty liver disease, NAFLD) has become one of the most prevalent chronic liver diseases worldwide, with its incidence continuously rising alongside the epidemic of metabolic disorders. AMP-activated protein kinase (AMPK), as a key regulator of cellular energy metabolism, influences multiple pathological processes associated with MASLD. This review systematically summarizes the regulatory roles of AMPK in lipid metabolism, inflammatory response, cell apoptosis, and fibrosis. Additionally, it discusses the latest developments of AMPK activators from preclinical to clinical studies, while analyzing the major challenges currently faced and potential strategies for resolution. A deeper understanding of AMPK regulatory mechanisms will contribute to the development of more effective therapeutic approaches for MASLD.

## 1 Introduction

Metabolic dysfunction-associated steatotic liver disease (MASLD; formerly known as non-alcoholic fatty liver disease, NAFLD) is a major etiological subtype of steatotic liver disease (1). According to the latest diagnostic consensus, MASLD is diagnosed in individuals with liver steatosis (defined histologically as the presence of lipid vacuoles in 5% or more of hepatocytes), at least one cardiometabolic risk factor (CMRF; including obesity, type 2 diabetes, dyslipidemia, and hypertension), and alcohol consumption less than 20-30 g per day (1, 2). In the wake of the public health epidemics of obesity and type 2 diabetes, MASLD has become one of the most common chronic liver diseases worldwide, with its prevalence continuously rising. Recent meta-analyses estimate a prevalence of approximately 38% in the adult population and around 65% in patients with type 2 diabetes (3, 4). The natural history of the disease shows that about 7-12% of patients with MASLD progress to cirrhosis or end-stage liver disease over periods of 7-20 years, and this progressive condition is closely associated with serious complications such as cardiovascular disease and extrahepatic malignancies (3, 5). Therefore, the development of new therapeutic targets and strategies is crucial for improving the prognosis of MASLD patients.

AMPK (adenosine monophosphate-activated protein kinase) serves as a central regulatory molecule in cellular energy metabolism and plays a crucial role in maintaining metabolic homeostasis (6). In the context of MASLD, hepatic AMPK activity is significantly reduced, while AMPK activation can ameliorate multiple pathological processes including lipid metabolism, inflammatory response, and fibrosis (7). Although preclinical studies have generally demonstrated the therapeutic potential of AMPK activators for MASLD, existing AMPK activators have not achieved the expected therapeutic efficacy in clinical trials, facing challenges in clinical translation (8). Moreover, the roles of AMPK at different pathological stages remain incompletely elucidated (7, 9). Therefore, developing therapeutic strategies that precisely modulate the AMPK signaling pathway through a deeper understanding of AMPK regulatory mechanisms holds significant importance for expanding treatment options for MASLD.

This review aims to systematically describe the expression and structural characteristics of AMPK, thoroughly discuss the regulatory mechanisms of AMPK in lipid metabolism, inflammatory response, cell apoptosis, and fibrosis in MASLD based on recent research advances, and summarize the progress and challenges of AMPK activators in preclinical and clinical studies, thereby providing theoretical basis for developing therapeutic strategies for MASLD.

## 2 Expression and structure of AMPK

Mammalian AMPK is a heterotrimeric complex composed of a catalytic α subunit and regulatory β and γ subunits. These subunits are encoded by PRKAA, PRKAB, and PRKAG genes, respectively. Each AMPK subunit has two to three isoforms (α1, α2, β1, β2, γ1, γ2, γ3), theoretically allowing the formation of 12 distinct trimeric AMPK complexes. These different AMPK subunit combinations exhibit variations in their regulatory characteristics and functional properties (10, 11). Analysis of human and mouse primary hepatocytes has revealed distinct species-specific expression patterns of AMPK subunits between these cell types. While the expression pattern of γ subunits is similar between the two cell types, significant differences exist in the expression of α and β subunits. Human hepatocytes predominantly express the α1 subunit with lower expression levels of the α2 subunit; both β1 and β2 subunits are expressed, with β2 being the predominant form. In contrast, mouse hepatocytes co-express α1 and α2 subunits with α2 being predominant; regarding β subunits, β1 is predominantly expressed while β2 expression is minimal (12).

**Figure 1:**
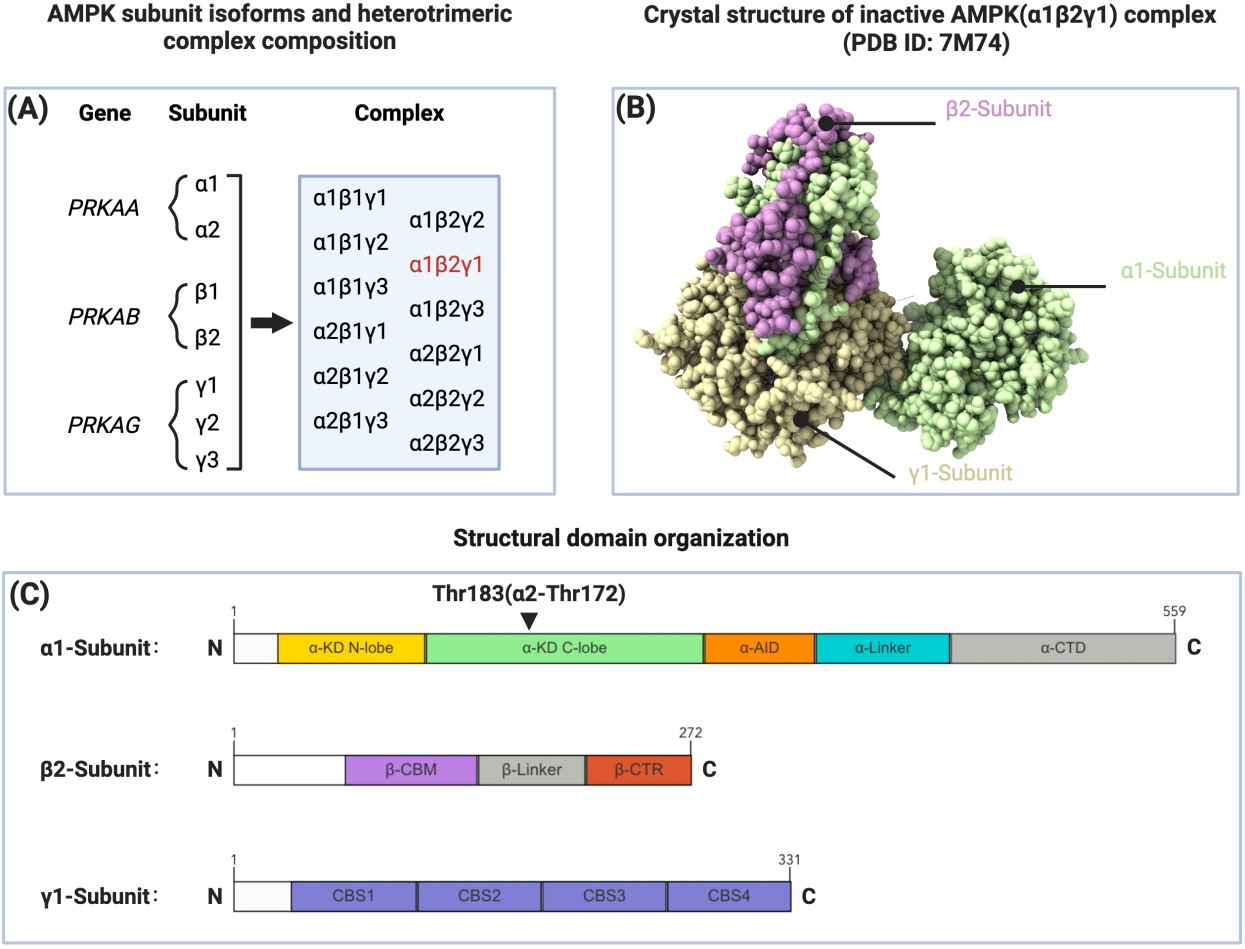
Subunit composition, structure, and domain organization of AMPK. **(A)** AMPK subunit isoforms and heterotrimeric complex composition. The PRKAA, PRKAB, and PRKAG genes encode α, β, and γ subunits respectively, which can form 12 distinct heterotrimeric combinations. The α1β2γ1 complex (highlighted in red) is considered the predominant AMPK isoform expressed in human liver. **(B)** Crystal structure of inactive AMPK(α1β2γ1) complex (PDB ID: 7M74). The three subunits are shown in different colors: α1-subunit (light green), β2-subunit (purple), and γ1-subunit (beige). **(C)** Structural domain organization of AMPK subunits. The α1-subunit (amino acids 1-559) consists of kinase domain (α-KD N-lobe and C-lobe), autoinhibitory domain (α-AID), linker region (α-Linker), and C-terminal domain (α-CTD). The β2-subunit (amino acids 1-272) contains carbohydrate-binding module (β-CBM), linker region (β-Linker), and C-terminal region (β-CTR). The γ1-subunit (amino acids 1-331) comprises four CBS domains (CBS1-4). Thr183(α2-Thr172) represents the key phosphorylation site of AMPK.

The α subunit contains an amino-terminal kinase domain (α-KD), followed by an autoinhibitory domain (α-AID), a linker region, and a C-terminal domain (α-CTD). The α-KD exhibits the classical structure of an N-lobe and C-lobe, with the Thr172 phosphorylation site located within the activation loop of the C-lobe being crucial for full AMPK activation. Notably, while this regulatory threonine residue is conventionally referred to as Thr172, the corresponding site in the human α1 subunit is actually Thr183 (9). The β subunit C-terminal regulatory domain (β-CTR) rigidly cross-links the α-CTD and γ subunit, forming the core of the regulatory module (9). The β subunit also includes a carbohydrate-binding module (β-CBM), which recognizes and binds carbohydrates such as glycogen and modulates AMPK activity (13). Additionally, constitutive myristoylation at the amino terminus of the β subunit is essential for AMP/ADP-mediated Thr172 phosphorylation (14, 15). The γ subunit contains four cystathionine-β-synthase (CBS) repeat sequences that form four potential ligand-binding sites, among which the competitive binding of adenosine monophosphate (AMP)/adenosine diphosphate (ADP) with adenosine triphosphate (ATP) at the CBS3 site represents the primary mechanism by which AMPK senses and responds to cellular energy levels to regulate its activity (16) (**Figure 1**).

## 3 AMPK regulation in MASLD

### 3.1 AMPK regulation of lipid metabolism in MASLD

MASLD is characterized by hepatic lipid homeostasis disruption, occurring when lipid input (including circulating free lipid uptake, dietary lipid absorption, carbohydrate-mediated *de novo* lipogenesis (DNL), and *de novo* cholesterol synthesis) exceeds lipid output capacity (fatty acid β-oxidation, very low-density lipoprotein-triglyceride (VLDL-TG) secretion, and cholesterol conversion to bile acids and secretion), resulting in gradual accumulation of neutral lipids (triglycerides and cholesterol esters) in hepatocytes (17). AMPK reduces hepatic lipid accumulation by inhibiting DNL and cholesterol synthesis while promoting fatty acid β-oxidation.

Glycolysis-derived pyruvate is converted to acetyl-CoA by the pyruvate dehydrogenase complex, and acetyl-CoA is further catalyzed by acetyl-CoA carboxylase (ACC) to form malonyl-CoA, an important DNL precursor (18). ACC is a classical downstream kinase of AMPK, which inhibits its activity through phosphorylation, thereby reducing DNL (19).

AMPK negatively regulates the activity and maturation of sterol regulatory element-binding protein (SREBP) family transcription factors through two pathways: inhibition of mammalian/mechanistic target of rapamycin complex 1 (mTORC1) and direct phosphorylation (20, 21). SREBP-1c, the primary hepatic SREBP-1 isoform, participates in DNL regulation by upregulating *acc* and *fasn* transcription (21–23). SREBP-2 specifically regulates the expression of cholesterol synthesis genes, including *hmgcr*, *hmgcs*, *fdps*, and *sqs* (23).

In fatty acid oxidation, cytosolic fatty acids first bind with CoA under the action of acyl-CoA synthetase to form acyl-CoA, which is then transferred to mitochondria by carnitine palmitoyltransferase 1 (CPT1) for β-oxidation. Since malonyl-CoA is an allosteric inhibitor of CPT1, AMPK promotes fatty acid β-oxidation by inhibiting ACC activity to reduce malonyl-CoA levels, thereby relieving CPT1 inhibition (24).

AMPK also participates in lipid metabolism through regulation of autophagy. In hepatic lipid droplet catabolism, autophagy-mediated lipid droplet degradation (lipophagy) works in concert with conventional cytosolic lipolysis: conventional lipolysis breaks down large lipid droplets into smaller ones, which are then further degraded through lipophagy (25). During this process, autophagosomes form and sequester lipid droplets, which then fuse with lysosomes where acidic lipases degrade triglycerides into fatty acids and promote β-oxidation (25).

AMPK, as a key regulator of autophagy, initiates autophagy by phosphorylating Unc-51 like autophagy activating kinase 1 (ULK1) and promotes autophagy by inhibiting mTORC1 through phosphorylation of either Tuberous sclerosis complex 2 (TSC2) or Regulatory-associated protein of mTOR (Raptor) (26). The mTORC1 complex (comprising mTOR, Raptor, mLST8, PRAS40, and Deptor) is a central hub in autophagy regulation that, under the influence of nutrients, energy, and growth factor signals, inhibits autophagy by suppressing Autophagy-related genes (ATG) proteins and lysosomal biogenesis (27). mTORC1’s phosphorylation of ULK1 disrupts the interaction between AMPK and ULK1 (26).

**Figure 2:**
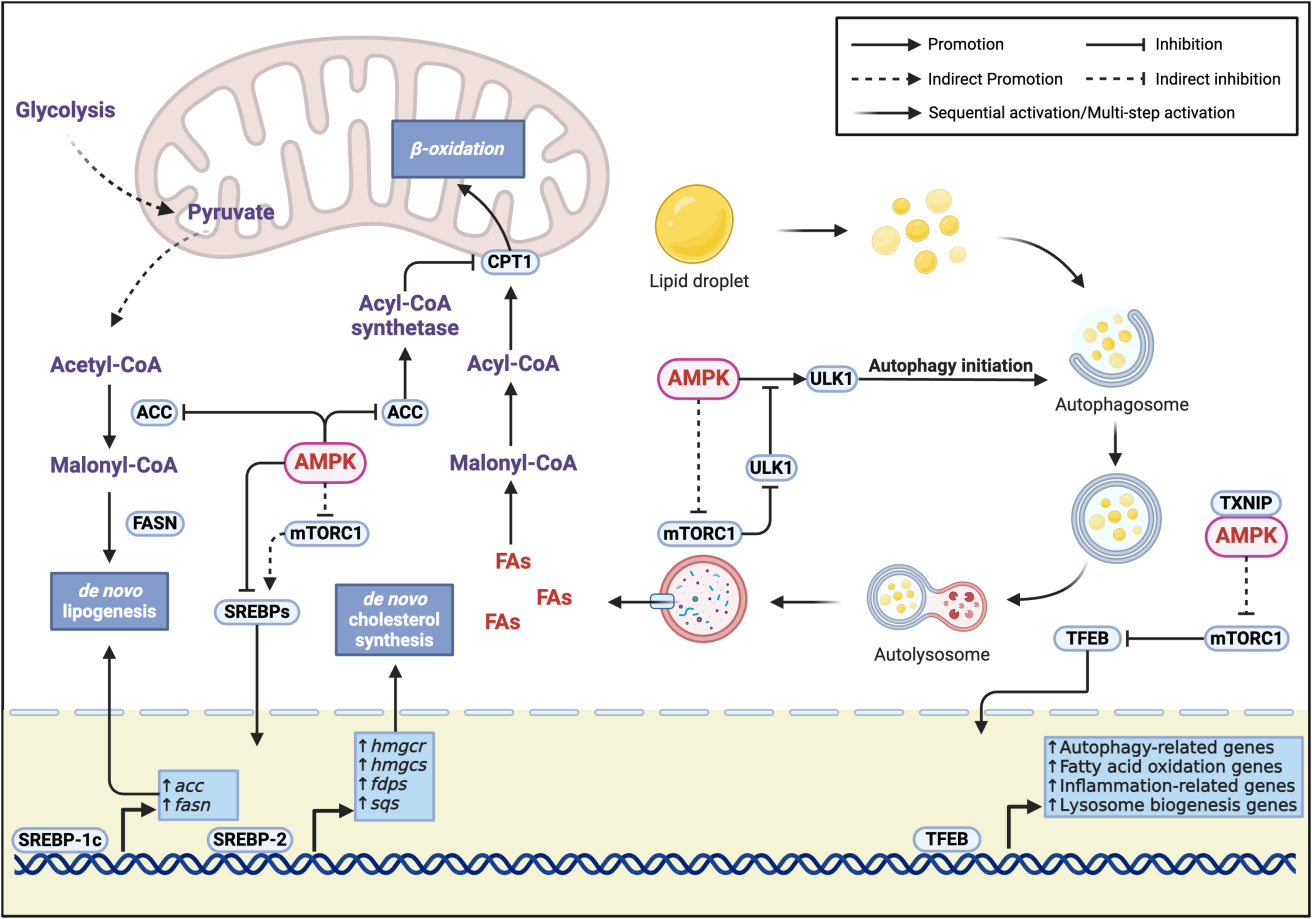
Molecular mechanisms of AMPK-regulated lipid metabolism. Lipid synthesis inhibition is achieved through three key pathways (1): Direct phosphorylation of ACC inhibits its activity, reducing the conversion of acetyl-CoA to malonyl-CoA, thereby suppressing the key step of *de novo* fatty acid synthesis (2); Dual inhibition of SREBP transcription factors through direct phosphorylation and mTORC1-dependent mechanisms, where SREBP-1c mainly regulates fatty acid synthesis-related genes (acc, fasn), and SREBP-2 specifically regulates cholesterol synthesis-related genes (hmgcr, hmgcs, fdps, sqs) (3); AMPK-mediated mTORC1 activity inhibition works synergistically with direct SREBP phosphorylation to effectively block the transcriptional activation of lipid synthesis-related genes. Lipid catabolism is regulated through two major pathways. AMPK inhibits ACC to reduce malonyl-CoA levels, relieving its inhibition on CPT1 and promoting fatty acid transport into mitochondria for β-oxidation. Additionally, AMPK promotes lipid breakdown through regulation of lipophagy, specifically by (1): Direct phosphorylation of ULK1 to initiate autophagosome formation (2); Inhibition of mTORC1 through phosphorylation of TSC2 or Raptor, relieving its phosphorylation-mediated inhibition of ULK1 and enhancing AMPK-ULK1 interaction (3); Promotion of TFEB nuclear localization through the TXNIP-mTORC1-TFEB axis, enhancing the transcription of autophagy-related genes and fatty acid oxidation genes, while improving inflammation and promoting lysosomal biogenesis, thereby strengthening lipophagy-mediated lipid droplet degradation.

Overall, the balance between AMPK and mTORC1 in autophagy regulation plays a crucial role in hepatic metabolism. In MASH mouse models, elevated hepatic expression of Thioredoxin-interacting protein/Vitamin D3 up-regulated protein 1 (TXNIP/VDUP1) interacts with AMPK to promote mTORC1 inactivation and Transcription factor EB (TFEB) nuclear translocation, thereby enhancing autophagy and fatty acid oxidation, leading to improvement in hepatic steatosis, inflammation, and fibrosis (28) (**Figure 2**).

Therefore, activated AMPK maintains hepatic lipid metabolic homeostasis through regulation of multiple downstream signaling pathways. In MASLD, hepatic AMPK activity is significantly reduced, though the underlying regulatory mechanisms remain incompletely understood (7). Recent studies have revealed several important regulatory molecules. In MASLD, decreased levels of circulating α-ketoglutarate (AKG), reduced expression of orosomucoid 2 (ORM2), and fibroblast growth factor 4 (FGF4) are closely associated with impaired AMPK activity (29–31). Additionally, upregulated hepatic Rho-associated coiled-coil containing protein kinase 1 (ROCK1) expression also suppresses AMPK activity (32). Notably, compensatory upregulation of the KISS1 (Kisspeptin 1)/KISS1R (KISS1 Receptor) system and cardiotrophin-1 (CT-1) may partially counteract the overall decrease in AMPK activity in MASLD by promoting AMPK activation (33).

A recent significant finding suggests the possible existence of AMPK-independent compensatory lipid regulatory mechanisms in the liver (12). Liver-specific AMPK-deficient mice maintained normal hepatic lipid homeostasis, and high-fat diet did not exacerbate MASLD development (12). However, this study also confirmed that reactivation of impaired AMPK could improve MASLD, consistent with most current research findings. Therefore, investigating the downstream mechanisms of AMPK regulation in hepatic lipid metabolism can provide theoretical basis for MASLD treatment through targeted AMPK activation, but may not explain the pathogenesis of MASLD.

### 3.2 AMPK regulation of inflammatory response in MASH

From a histological perspective, the MASLD disease spectrum encompasses metabolic dysfunction-associated steatosis of the liver (MASL) and metabolic dysfunction-associated steatohepatitis (MASH), with patients potentially developing progressive liver fibrosis, ultimately leading to cirrhosis and/or hepatocellular carcinoma (HCC) (3, 34). The pathogenesis of MASH involves three interconnected pathological processes: chronic inflammatory response mediated by pro-inflammatory mediators [such as TNF-α (Tumor Necrosis Factor-alpha), IL-6 (Interleukin-6), IL-1β (Interleukin-1 beta), ROS (Reactive Oxygen Species)], programmed hepatocyte death, and fibrosis progression mediated by hepatic stellate cell activation (35).

AMPK, as a crucial kinase widely expressed in various hepatic cells, exerts multiple protective effects when activated. It maintains hepatocyte energy balance, suppresses pro-inflammatory mediator production, counteracts hepatocyte injury through regulation of mitochondrial function and cell death pathways, and alleviates liver fibrosis by inhibiting hepatic stellate cell activation (36–38). Recent studies have demonstrated that in a MASH model induced by choline-deficient high-fat diet (CD-HFD), hepatic AMPK deficiency exacerbates hepatocyte death and liver injury (38). Therefore, AMPK likely plays a significant role in the progression from MASLD to MASH.

#### 3.2.1 AMPK regulation of pro-inflammatory mediators in MASH

In the pathological progression of MASH, excessive lipid accumulation and lipotoxicity trigger a cascade of events: damaged hepatocytes release damage-associated molecular patterns (DAMPs), which subsequently activate Kupffer cells (KCs) and infiltrating macrophages. These activated immune cells produce large quantities of pro-inflammatory mediators, further exacerbating hepatocyte injury, thus creating a vicious cycle (39, 40).

This pathological dialogue between hepatocytes and immune cells constitutes the core of the inflammatory response in MASH (41). AMPK exerts significant inhibitory effects on this process by regulating the production of pro-inflammatory mediators in these cells.

##### 3.2.1.1 AMPK regulation of pro-inflammatory mediator production in hepatocytes

Enhanced AMPK signaling in hepatocytes suppresses cytokine expression. Studies in HFD mouse models have demonstrated that AMPK inhibits the expression of TNF-α, IL-1β, and IL-6 by activating its downstream target Sirtuin1 (SIRT1), which reduces p65 acetylation levels (36).

Mitochondrial dysfunction is a crucial factor in the inflammatory response of MASH. Rodent model studies indicate that mitochondrial function may temporarily increase adaptively during the MASL stage but becomes significantly impaired during the MASH stage due to sustained lipotoxicity (42). Excessive free fatty acids lead to electron transport chain (ETC) overload, increasing ROS generation. These ROS not only directly damage hepatocytes but also act as pro-inflammatory mediators activating downstream inflammatory signaling pathways, creating a vicious cycle of inflammatory response (42, 43).

Mitochondria are highly dynamic organelles whose structure and number constantly change in response to cellular energy demands and physiological stimuli, with AMPK being one of the classical regulators of this process (44). Studies have shown that AMPK can sense mitochondrial dysfunction through the upstream ROS-CaMKK2 signaling pathway and participates in regulating multiple key aspects of mitochondrial quality control (45–47).

Specifically, AMPK can upregulate Peroxisome proliferator-activated receptor gamma coactivator 1-alpha (PGC1α) either directly through phosphorylation or indirectly through p38MAPK (p38 Mitogen-Activated Protein Kinase), SIRT1, and HDAC5 (Histone Deacetylase 5)-mediated pathways, thereby promoting mitochondrial biogenesis-related gene transcription and mitochondrial DNA replication, enhancing mitochondrial content and oxidative phosphorylation capacity (6, 48). Regarding mitochondrial dynamics regulation, AMPK participates in mitochondrial fission by phosphorylating Mitochondrial Fission Factor (at Ser155 and Ser173 sites), promoting DRP1 recruitment (49).

Recent studies have revealed that under mitochondrial stress conditions, the AMPK-FNIP1 (Folliculin Interacting Protein 1) signaling axis demonstrates an integrated regulatory role in mitochondrial quality control, unveiling a temporal response mechanism to mitochondrial damage (50). AMPK phosphorylation of FNIP1 leads to nuclear translocation of TFEB/TFE3 (Transcription Factor E3), prioritizing the expression of lysosomal and autophagy-related genes for damaged mitochondria clearance, while simultaneously inducing transcription of NT-PGC1α (an N-terminal truncated isoform of PGC1α with enhanced protein stability). Once NT-PGC1α protein accumulates to sufficient levels, it activates new mitochondrial generation through the NT-PGC1α-ERRα (Estrogen-Related Receptor alpha) transcriptional axis, completing mitochondrial renewal (50). This temporal regulatory pattern not only ensures efficient mitochondrial renewal but also reflects cellular energy utilization economy.

AMPK precisely coordinates the selective autophagy of functional and damaged mitochondria through two independent mechanisms. AMPK phosphorylates ULK1 at Ser556 and Ser694 sites to promote 14-3-3 protein binding, inhibiting NIX (neighbor of BRCA1 gene 1 protein X)-dependent functional mitochondrial autophagy, while simultaneously directly phosphorylating Parkin at Ser108 to activate PINK1 (PTEN-induced kinase 1)/Parkin-dependent clearance of damaged mitochondria (51–53). Therefore, AMPK may improve hepatocyte mitochondrial dysfunction through these quality control mechanisms and suppress NLRP3 (NOD-Like Receptor Family Pyrin Domain Containing 3) inflammasome activation and IL-1β release, reducing inflammatory responses in MASH (46, 54). Additionally, AMPK enhances antioxidant capacity by activating Nrf2 (Nuclear Factor Erythroid 2-Related Factor 2), protecting hepatocytes from oxidative damage and subsequent pro-inflammatory mediator secretion (29).

##### 3.2.1.2 AMPK regulation of pro-inflammatory mediator production in macrophages

As MASLD/MASH progresses, embryonic-derived resident Kupffer cells (KCs) in the liver are gradually replaced by circulating recruited macrophages, including monocyte-derived macrophages (Mo-Ms) and newly formed KCs differentiated from circulating monocytes. This replacement process leads to changes in the hepatic macrophage pool composition, with newly recruited macrophages exhibiting pro-inflammatory phenotypes, further secreting inflammatory mediators such as TNF and IL-1β, and promoting disease progression (41).

AMPK activation in hepatic macrophages significantly suppresses inflammatory factor production. Li et al. (55) demonstrated that in RAW264.7 cells and KCs, AMPK reduces pro-inflammatory mediator secretion by inhibiting the Nuclear Factor of Kappa Light Polypeptide Gene Enhancer in B-cells Inhibitor Alpha (IκBα)-NF-κB pathway and the iNOS/NO system (56). Furthermore, Park et al. (57) discovered in a high-fat diet mouse model that metformin activated the AMPK-SIRT1-Tristetraprolin (TTP) signaling axis in KCs, thereby inhibiting TNF-α production.

In circulating recruited macrophages, the β1 subunit of AMPK plays a crucial role in functional regulation. Studies have shown that β1 deficiency leads to reduced AMPKα1 activity, resulting in decreased mitochondrial content and impaired fatty acid oxidation, promoting macrophages to acquire an M1 pro-inflammatory phenotype and increased sensitivity to fatty acid-induced inflammatory responses (58, 59). Recent studies have revealed that circulating monocytes in MASH patients undergo significant metabolic reprogramming, characterized by a shift in energy metabolism from oxidative phosphorylation to glycolysis, accompanied by enhanced glycolysis and mitochondrial respiration, mitochondrial dysfunction, and oxidative stress (60). Mechanistic studies have shown that AMPK activity suppression in Mo-Ms can enhance mitochondrial respiration through activation of the mTOR-PGC1α signaling pathway, promoting ROS generation and subsequently exacerbating inflammatory responses (60).

#### 3.2.2 AMPK inhibits MASH-related liver fibrosis in HSCs

During MASH, hepatic stellate cells (HSCs) respond to various immune mediators, including pro-inflammatory cytokines and chemokines, transforming from a quiescent state to a myofibroblast-like activated state. Activated HSCs synthesize and secrete large amounts of extracellular matrix (ECM) components, ultimately leading to liver fibrosis (61). HSCs have been identified as the primary effectors driving liver fibrosis (62).

*In vitro* studies of activated primary mouse HSCs have shown increased AMPKα1 activity (63). Further pharmacological activation of AMPK effectively inhibits HSC proliferation (63–65). Additionally, soluble guanylate cyclase (sGC) is expressed in HSCs and their derived fibroblasts but not in hepatocytes. In MASH mouse models, the sGC stimulator Praliciguat significantly improved liver inflammation and fibrosis through activation of the sGC-cyclic guanosine monophosphate (cGMP)-AMPK signaling pathway (37). These findings demonstrate that pharmacological activation of AMPK effectively suppresses the fibrotic phenotype both in cultured HSCs and in preclinical models of MASH.

Studies have shown that AMPK maintains HSC homeostasis and inhibits their pathological proliferation by regulating antioxidant stress responses and cell cycle progression (64, 65). On one hand, AMPK blocks protein kinase B (AKT/PKB) signaling pathway activation by inducing antioxidant enzyme expression and inhibiting nicotinamide adenine dinucleotide phosphate (NADPH) oxidase-dependent ROS generation. On the other hand, AMPK suppresses HSC proliferation by upregulating p27 and p21 expression, possibly mediated through p53 phosphorylation (64). Furthermore, AMPK impedes HSCs proliferation and fibrosis progression by inhibiting NF-κB and mTOR pathway activities (65).

During HSCs activation, Transforming growth factor-beta (TGF-β) promotes collagen transcription through its signaling substrates Mothers against decapentaplegic homolog 2/3 (SMAD2/3) (66). Lim et al. (66) demonstrated that AMPK inhibits transdifferentiation and fibrogenic gene expression by interfering with the TGF-β/SMAD pathway. Pharmacological activation of AMPK using 5-Aminoimidazole-4-carboxamide-1-β-D-ribofuranoside (AICAR), metformin, or adiponectin competitively binds to transcriptional coactivator p300, inhibiting p300-mediated SMAD3 acetylation and reducing collagen gene expression (66). Recently, Hall et al. (37) discovered that AMPK activated by the NO-sGC-cGMP signaling pathway suppresses TGF-β signaling and fibrosis progression by upregulating SMAD7 expression, and this mechanism operates independently of SMAD2/3 (37). This indicates that the AMPK pathway in HSCs exhibits specific downstream molecular responses to different upstream signals. Further investigation of these molecular mechanisms may help understand how AMPK mediates adaptive regulation of HSCs in different physiological or pathological environments.

During HSCs activation, intracellular pH (pHi) significantly increases due to enhanced proton efflux mediated by Vacuolar-type H+-ATPase (v-ATPase). v-ATPase is a multi-subunit complex that regulates pHi through ATP-dependent H+ transmembrane transport. Studies have shown that pharmacological activation of AMPKα1 inhibits v-ATPase activity, effectively reducing the pro-fibrotic phenotype of HSCs (67).

#### 3.2.3 AMPK suppresses hepatocyte apoptosis and liver injury

**Figure 3:**
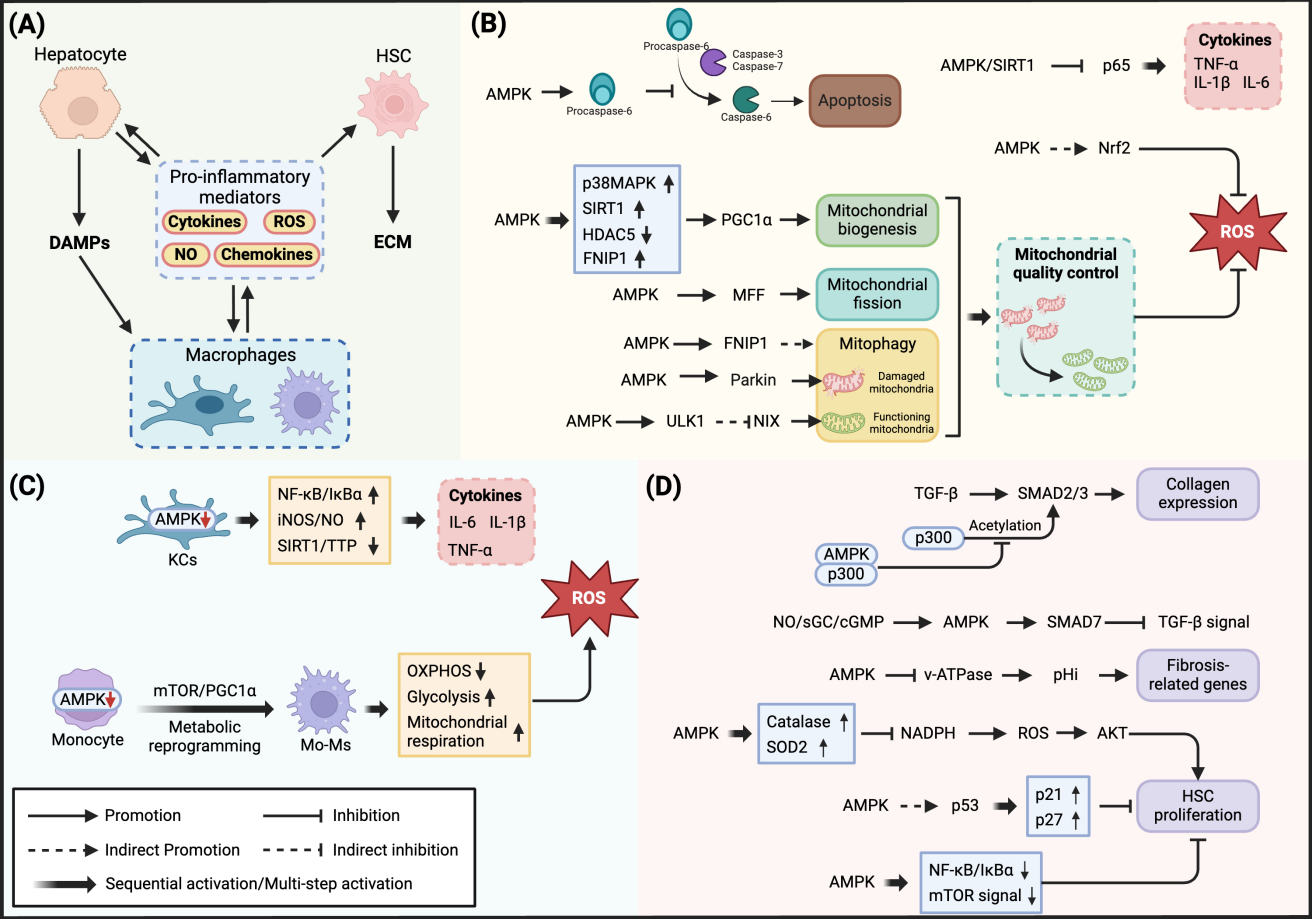
Regulatory mechanisms of AMPK in MASH-associated inflammatory response. **(A)** Intercellular pro-inflammatory mediator network in MASH pathology: Hepatocyte injury releases DAMPs, triggering macrophage activation and production of pro-inflammatory mediators (cytokines, ROS, NO, and chemokines), which amplify inflammatory responses and induce hepatic stellate cell activation. **(B)** AMPK regulation of inflammatory response in hepatocytes: In hepatocytes, AMPK inhibits inflammatory cytokine expression through SIRT1-mediated p65 deacetylation. Meanwhile, AMPK regulates mitochondrial quality through multiple levels: promoting mitochondrial biogenesis via p38MAPK/SIRT1/HDAC5/FNIP1-PGC1α axis; regulating mitochondrial dynamics through MFF; coordinating mitophagy via FNIP1-TFEB/TFE3 and Parkin/ULK1-NIX dual pathways. Additionally, AMPK enhances antioxidant defense by activating Nrf2 and inhibits apoptotic cascade initiation through Procaspase-6 phosphorylation. **(C)** AMPK regulation of inflammatory response in macrophages: In Kupffer cells, AMPK reduces pro-inflammatory mediator production by inhibiting NF-κB/IκBα and iNOS/NO signaling pathways while activating the SIRT1-TTP axis. In monocyte-derived macrophages, decreased AMPK activity leads to mTOR/PGC1α pathway activation, resulting in metabolic reprogramming and mitochondrial dysfunction, increased ROS production, and exacerbated inflammatory response. **(D)** AMPK regulation of fibrosis in hepatic stellate cells: Competitively binding with p300 to inhibit SMAD2/3-mediated collagen expression; suppressing TGF-β signaling through NO/sGC/cGMP-mediated SMAD7 upregulation; reducing cellular pH by inhibiting v-ATPase activity; suppressing ROS-AKT signaling pathway through antioxidant enzyme upregulation; inhibiting HSC proliferation via p53-p21/p27 axis and NF-κB/mTOR pathway.

Hepatocyte injury and death can be triggered by lipotoxicity-induced mitochondrial dysfunction and endoplasmic reticulum stress, as well as inflammatory signaling pathways activated by pro-inflammatory mediators. Extensive hepatocyte death leads to persistent liver injury, promoting connective tissue deposition and inter-regional fibrotic connections, ultimately progressing to cirrhosis. Apoptosis is one of the primary forms of hepatocyte death in MASH (68). The apoptotic process is mediated by the activation of Cysteine-dependent aspartate-specific proteases (caspases), where initiator caspases such as caspase-8 and caspase-9 initiate the apoptotic cascade, while effector caspases including caspase-3, caspase-6, and caspase-7 execute cell apoptosis (69). Studies have shown that during the progression from MASL to MASH, reduced AMPK activity is closely associated with apoptosis and tissue damage (38). In metabolically healthy liver, AMPK inhibits caspase-3 and caspase-7-mediated cleavage and activation of procaspase-6 through phosphorylation at Ser257. However, under MASH conditions, as AMPK activity decreases, the inhibition of procaspase-6 is relieved, leading to its cleavage and release of active caspase-6. Caspase-6 subsequently cleaves BH3-interacting domain death agonist (Bid), releasing cytochrome c from mitochondria, thereby forming a feed-forward loop that continuously induces cell apoptosis and exacerbates liver injury (38) (**Figure 3**) (**Table 1**).

**Table 1:** AMPK-mediated regulatory effects in MASLD.

Regulatory Function	Mechanism	Biological Effects
Metabolic Regulation			
Lipid synthesis inhibition	DNL inhibition (19, 21–23) Cholesterol synthesis inhibition (23)	Reduction of lipid accumulation
Lipid degradation promotion	Promotion of fatty acid oxidation (24) Enhanced lipophagy (26–28)	Enhancement of lipid consumption
Inflammation Regulation			
Hepatocyte-derived inflammation inhibition	Transcriptional suppression of cytokine expression (36) Mitochondrial quality control (6, 47–53) Oxidative stress defense (29)	Reduction of pro-inflammatory mediator production
Macrophage-derived inflammation inhibition	KC function regulation (56, 57) Mo-MΦ differentiation inhibition (58–60)	Suppression of pro-inflammatory phenotype transformation Reduction of pro-inflammatory mediator production
Fibrosis Regulation			
HSC activity inhibition	Proliferation inhibition (63–65) Metabolic homeostasis maintenance (64, 65, 67)	Inhibition of HSC activation and proliferation
Fibrogenic factor inhibition	TGF-β pathway regulation (37, 66) Fibrotic gene suppression (66)	Reduction of ECM synthesis and deposition
Cell Death Regulation			
Apoptosis inhibition	Caspase-6 regulation (38)	Reduction of cell apoptosis

## 4 AMPK activation and its application in MASLD treatment

Although the FDA approved Rezdiffra (resmetirom) in 2024 for treating non-cirrhotic MASH in adults, treatment options remain limited. As discussed above, AMPK plays a crucial role in MASLD development and progression, representing a promising therapeutic target. AMPK regulates cellular metabolism through diverse signaling input and output networks, and its activation mechanisms primarily include typical nucleotide sensing pathways, upstream kinase regulation, and post-translational modifications (70). Elucidating the regulatory characteristics of these activation mechanisms in MASLD will facilitate the development of more precise AMPK-targeted therapeutic strategies.

### 4.1 Classical nucleotide sensing activation mechanism

When cellular energy levels decrease, elevated AMP/ATP ratio leads to AMP replacing ATP at the CBS3 site of the AMPK γ subunit, inducing an active conformational change in AMPK. In this conformation, dephosphorylation of Thr172 in the activation loop is inhibited, and phosphorylation activation is completed under the catalysis of upstream kinases (9). As the primary source of cellular ATP, mitochondrial functional status directly affects AMPK activity. Under normal conditions, the proton gradient generated by the mitochondrial respiratory chain produces ATP through ATP synthase. Mitochondrial uncoupling allows protons to bypass ATP synthase and flow back directly, reducing ATP generation and releasing heat. Therefore, both inhibition of the respiratory chain and promotion of mitochondrial uncoupling can activate AMPK by increasing the cellular AMP/ATP ratio (6).

Considering that AMPK is not the sole responder to cellular energy stress, relying solely on the nucleotide-sensing mechanism that regulates cellular energy levels may not be the optimal choice for developing precise AMPK-targeting drugs (71–73). Nevertheless, several clinical drugs involving energy-sensing mechanisms for AMPK regulation have been validated in other diseases, and their repurposing value in MASLD treatment remains worthy of attention (74).

Metformin, widely used clinically for treating type 2 diabetes, functions by inhibiting the mitochondrial electron transport chain, reducing mitochondrial respiration, thereby activating AMPK (74). Although its efficacy in treating MASLD and MASH and potential hepatotoxicity remain controversial, recent evidence supports its therapeutic potential (57, 75–78). However, Liu et al. (79) found that functional leptin receptor is essential for metformin’s therapeutic efficacy, suggesting that long-term metformin treatment might promote MASLD progression in leptin-insensitive individuals, limiting its clinical application scope.

Furthermore, ezetimibe, a medication for treating hypercholesterolemia, has shown significant improvement in hepatic steatosis and inflammation scores in MASH patients after 24 months of treatment in clinical studies (80). Although ezetimibe had not previously been reported to improve its original indication through the AMPK pathway, Kim et al. (81) discovered that it improves MASH by inducing autophagy through activation of the AMPK-TFEB pathway. Studies have shown that ezetimibe’s ability to reduce hepatocyte ATP levels might be one of its key mechanisms for AMPK activation, though the specific molecular mechanisms require further investigation (81). Similarly, low-dose sorafenib (one-tenth of the clinical HCC treatment dose) can safely and effectively inhibit MASH progression in mice and monkeys. Unlike its kinase-targeting mechanism in HCC, at this dose, sorafenib primarily achieves therapeutic effects through inducing mitochondrial uncoupling and activating AMPK (82).

### 4.2 Upstream kinase activation mechanism

#### 4.2.1 LKB1-AMPK activation mechanism

Liver kinase B1 (LKB1) is the primary upstream kinase of AMPK, which forms an active complex with STRAD and MO25, triggering nucleocytoplasmic shuttling and conformational changes, ultimately activating AMPK. Unlike other kinases, LKB1 possesses constitutive activity, a characteristic that may provide a degree of assurance for AMPK’s rapid response to elevated AMP concentrations. Although LKB1 has basal enzymatic activity, its functional strength and regulatory efficiency remain subject to multiple fine-tuned regulations (83).

Various post-translational modifications regulate the LKB1-AMPK pathway by affecting LKB1’s kinase activity, subcellular localization, and expression stability. Protein kinase A (PKA) can enhance LKB1 signaling efficiency through phosphorylation; studies have shown that dehydroepiandrosterone (DHEA) and hyperforin (HP) can improve MASLD animal models by activating the PKA-LKB1-AMPK pathway (84, 85). Conversely, Fyn tyrosine kinase (Fyn) restricts LKB1 to the nucleus through phosphorylation at Tyr261/365 sites (83). In MASLD, increased hepatocyte Cluster of differentiation 36 (CD36) palmitoylation leads to the formation of CD36/Fyn/LKB1 complexes, where Fyn phosphorylates LKB1, promoting its nuclear translocation and subsequently inhibiting AMPK activity (86). Under MASH conditions, oxidative stress triggers increased MAPK phosphatase-1 (MKP1) expression, leading to p38 MAPK dephosphorylation in the nucleus. This process inhibits the phosphorylation sites required for LKB1 nuclear export, causing LKB1 retention in the nucleus. Consequently, cytoplasmic AMPK activation is suppressed, ultimately leading to hepatocyte death and MASH progression (87).

LKB1 contains multiple acetylation sites on its lysine residues, and SIRT1 regulates LKB1 stability through Lys48 site deacetylation, promoting its interaction with E3 ubiquitin ligase and mediating LKB1 degradation through the proteasome (83). Numerous preclinical studies indicate that the SIRT1-LKB1-AMPK pathway is an important therapeutic target for MASLD. Transcription factor p53 (88), thyrotropin (22), and natural compounds such as berbamine (BBM) (89) and apple polyphenol extract (APE) (90) all exert therapeutic effects through this pathway.

LKB1 undergoes farnesylation at the Cys430 cysteine residue within its conserved CAAX sequence, a post-translational modification crucial for LKB1’s membrane localization and function (83). Similarly, myristoylation of the AMPK β subunit enhances its membrane-binding capacity. These fatty acid modifications may influence LKB1-AMPK signaling pathway activity by regulating protein spatial distribution through membrane localization (91). Studies have shown that under high-fat diet (HFD) conditions, downregulation of geranylgeranyl diphosphate synthase (GGPPS) in mouse hepatocytes leads to enhanced LKB1 farnesylation. This change triggers metabolic reprogramming through upregulation of the LKB1-AMPK axis, accelerating glycolysis and ultimately exacerbating hepatic inflammation and fibrosis (92).

The binding of LKB1 to AMPK involves the scaffolding function of AXIN, where cytoplasmic AMP drives AXIN (Axis inhibition protein) to directly tether LKB1 for AMPK phosphorylation (93). Studies have shown that adenovirus-based knockdown of AXIN in mouse liver impairs AMPK activation and exacerbates fasting-induced hepatic steatosis (93). Recently, researchers discovered another scaffold protein, SCO1 (Synthesis of cytochrome c oxidase 1) (94). As a copper-sensing molecular chaperone, SCO1 can activate AMPK through the formation of SCO1-LKB1-AMPK complexes. Importantly, ceruloplasmin deficiency can restore hepatic copper balance in obese mice and improve MASLD progression by enhancing mitochondrial biogenesis and fatty acid oxidation (94).

Beyond these regulatory mechanisms, studies have found that certain natural compounds can directly bind to LKB1 to modulate its activity (95). Furthermore, regulation of STK11 (the gene encoding LKB1) expression at the genetic level can also influence LKB1-AMPK pathway activity (96). These diverse regulatory mechanisms provide rich potential targets for developing MASLD therapeutic strategies.

#### 4.2.2 CaMKK2-AMPK activation mechanism

CaMKK2 (Calcium/calmodulin-dependent protein kinase kinase 2) is another important upstream kinase of AMPK, with its activity primarily regulated by calcium signaling. Cells maintain extremely low cytoplasmic calcium concentrations through active transport by plasma membrane and endoplasmic reticulum Ca^2+^ ATPases (97). One common calcium signaling pathway in mammalian cells is initiated when ligands bind to G protein-coupled receptors or receptor tyrosine kinases (RTK), leading to phospholipase C-catalyzed generation of inositol 1,4,5-trisphosphate (IP3). IP3 then binds to IP3 receptors (IP3Rs) on the endoplasmic reticulum, opening these channels and releasing stored calcium into the cytoplasm (98). When cytoplasmic calcium concentration increases, the Ca^2+^/calmodulin complex interacts with CaMKK2, inducing conformational changes and activating AMPK (99).

The IP3Rs-CaMKK2 signaling axis represents an important pathway for targeting AMPK to improve MASLD. Studies have shown that hepatokine FGF4 activates the CaMKK2-AMPK-Caspase 6 signaling axis through the classical PLCγ-IP3-IP3Rs cascade initiated by RTK family receptor FGFR activation, thereby exerting therapeutic effects on MASH (31). Zhou et al. (30) discovered that hepatokine ORM2 directly binds to membrane IP3R2 through autocrine/paracrine mechanisms, subsequently activating the CaMKK2-AMPK pathway to regulate lipid metabolism and improve hepatic steatosis and inflammation in mice.

The interaction between CaMKK2 and AMPK also involves scaffold proteins. Research has shown that exercise can induce hepatic expression of cysteine dioxygenase type 1 (Cdo1) (100). Cdo1 functions as a scaffold protein promoting CaMKK2-AMPK binding, forming a CaMKK2-Cdo1-AMPK trimolecular complex that enhances AMPK activation (100). This study provides new insights into explaining the metabolic benefits of exercise in the liver. Besides Cdo1, STIM2 has been identified as another scaffold protein facilitating CaMKK2-AMPK interaction, though its role in MASLD pathogenesis requires further investigation (101).

Among the 22 identified phosphorylation sites of CaMKK2, 9 sites have regulatory functions. These sites are regulated by various upstream kinases including PKA, Cyclin-dependent kinase 5 (CDK5), Glycogen synthase kinase 3 (GSK3), and AMPK (99). Notably, AMPK exerts negative feedback regulation by phosphorylating CaMKK2 at Thr145, limiting its basal activity while not affecting the maximal activity mediated by Ca²⁺/calmodulin (102).

### 4.3 Post-translational modifications beyond Thr172

Beyond the phosphorylation of AMPK α subunit at Thr172, phosphorylation modifications at other sites also play significant roles in hepatic metabolic regulation. Research by Gao et al. (103) demonstrated that in SD rat models, high-fat diet inhibits AMPK activity by promoting AKT-mediated phosphorylation of AMPKα1 at Ser487. Furthermore, Zhao et al. (104) discovered that TANK-binding kinase 1 (TBK1), a non-canonical IKK family member in adipocytes, can phosphorylate AMPKα1 at Ser459 and Ser476, thereby inhibiting AMPK Thr172 phosphorylation and activation. These sites, located near the β-subunit interaction domain and ST loop of the α subunit, are conserved between AMPKα1 (PRKAA1) and AMPKα2 (PRKAA2) isoforms and across species from Drosophila to humans (104). Recently, Luo et al. (105) showed that myeloid differentiation factor 2 (MD2) contributes to MASLD pathogenesis through the TBK1-AMPK/SREBP1 and lipid metabolism pathways.

Ser108 on the AMPK β1 subunit is a cis-autophosphorylation site maintained at low levels under basal conditions. β1 is specifically phosphorylated by the autophagy-initiating kinase ULK1, a feature absent in β2. Studies using mice with AMPK β1 Ser108 loss-of-function mutations have shown that phosphorylation at this site is crucial for stimulating mitochondrial biogenesis and mitophagy (106). However, mouse studies have also revealed that although AMPK β1 Ser108 phosphorylation levels increase under high-fat diet conditions, this modification is not essential for regulating whole-body fatty acid oxidation. This finding suggests that simply upregulating phosphorylation at this site may not be an ideal strategy for MASLD treatment (106).

O-linked β-N-acetylglucosamine modification (O-GlcNAcylation) is a unique form of O-glycosylation occurring in the cytoplasm and nucleus. This dynamic and reversible modification, catalyzed by O-GlcNAc transferase (OGT), adds a single N-acetylglucosamine to serine and threonine residues. These sites are often also targets for phosphorylation (107). Studies have shown that O-GlcNAcylation inhibits AMPK Thr172 phosphorylation and reduces its activity (108). Recent research indicates that sodium-glucose cotransporter 2 (SGLT2) inhibitors can improve MASH by reducing SGLT2 O-GlcNAcylation levels, thereby promoting AMPK activity and autophagy (109).

N-terminal myristoylation (N-myristoylation) is a widespread post-translational modification in eukaryotic cells, involving the covalent attachment of myristic acid (C14:0) to the N-terminal glycine of proteins (110). AMPK was among the first proteins discovered to have this modification, with Gly2 sites on both β1 and β2 subunits being myristoylated by N-myristoyltransferase (NMT). Research has shown that the absence of AMPK β subunit myristoylation leads to increased Thr172 phosphorylation and activity levels, potentially due in part to reduced interaction with phosphatases. In high-fat diet-induced animal models, myristoylation deficiency improved insulin sensitivity by reducing hepatic lipid accumulation and adipose tissue proliferation (111).

Ubiquitination is an important post-translational modification that regulates protein stability, activity, and cellular localization through a cascade reaction involving E1 activating enzymes, E2 conjugating enzymes, and E3 ligases, which covalently attach ubiquitin molecules to substrate proteins (112). Studies have found that the E3 ubiquitin ligase Makorin Ring Finger Protein 1 (MKRN1) can bind to AMPK and mediate its ubiquitination-dependent degradation. MKRN1 deficiency leads to enhanced AMPK activity, promoting glucose utilization and inhibiting lipid accumulation (113).

### 4.4 ADaM site binding

The Allosteric Drug and Metabolite (ADaM) site is a deep binding pocket between the β-CBM and kinase domain N-lobe, whose stability depends on Ser108 phosphorylation on β-CBM (9). When activators bind to the ADaM site, they induce allosteric activation of AMPK, stabilizing the activation loop structure and protecting pThr172 from dephosphorylation (9). ULK1-specific phosphorylation of β1 Ser108 enhances the affinity of β1-containing AMPK complexes for ADaM site activators (11). Several ADaM site activators have been identified and developed, including A-769662, salicylate, PXL770, and PF-06409577 (8, 114–116). Recent research has revealed that long-chain fatty acyl-CoAs (LCFA-CoAs, C12-C24) are natural ligands for the ADaM site. Although the sequences surrounding Ser108 are relatively conserved between AMPK β1 and β2, LCFA-CoAs only activate β1-containing AMPK complexes when Ser108 is phosphorylated, while β2 complexes are insensitive (106).

Various ADaM site activators have shown MASLD-improving effects in preclinical studies. Given that human hepatocytes predominantly express the AMPK β2 subunit, clinical development of these drugs requires special attention to their isoform selectivity. Recent cell-free assay studies showed that PXL770 has significant selectivity for β1 complexes (117). Nevertheless, both PXL770 and PF-06409577 demonstrated MASLD-improving effects in humans and non-human primates, suggesting therapeutic efficacy can be achieved even with highly selective β1 complex activation (8, 116).

PXL770 is the first and currently only direct AMPK activator to enter clinical trials for MASLD (118). In a phase 2a clinical trial involving 120 patients, PXL770 showed good tolerability (8). Regarding efficacy, compared to the placebo group (-1.1%), a total daily dose of 500mg reduced liver fat content by approximately 14% at 12 weeks, and although not reaching statistical significance, improvements in some metabolic parameters were observed. This is consistent with AMPK’s molecular mechanism of inhibiting hepatic lipogenesis and promoting fatty acid oxidation. Additionally, non-parametric sensitivity analysis showed statistical significance in the 500mg once-daily group (p=0.039). Moreover, more pronounced therapeutic effects were observed in the type 2 diabetes patient subgroup (8). This provides preliminary clinical evidence for the potential value of AMPK activation strategies in MASLD treatment, warranting further validation in larger clinical trials.

### 4.5 Other mechanisms

As AMPK research progresses, related mechanisms continue to be updated. Emerging evidence suggests that beyond the aforementioned mechanisms, AMPK’s role in hepatic metabolic regulation also involves nucleocytoplasmic localization regulation and interactions with various autophagy-related proteins.

Research by Jang et al. (119) demonstrated that Thyroid hormone receptor-associated protein 3 (Thrap3) can directly interact with the AMPK α subunit through its C-terminal region. This interaction confines AMPK to the nucleus, thereby negatively regulating the AMPK/autophagy axis in MASLD.

Park et al. (28) showed that in FFA-induced hepatocytes, TXNIP enhances AMPK activity through direct binding to the AMPK α subunit, subsequently triggering autophagy and fatty acid oxidation, thereby inhibiting MASH development. Furthermore, Lee et al. (120) discovered that SQSTM1 binds to AMPK through its PB1 domain while also interacting with ULK1, forming an endogenous SQSTM1-AMPK-ULK1 trimolecular complex. The formation of this complex is significantly enhanced under lipotoxic conditions. Mechanistic studies further revealed that SQSTM1 can also bind to SESN2 (sestrin 2), a known regulator of AMPK activation that promotes AMPK activation through multiple mechanisms. In this way, the formation of the SQSTM1-AMPK-ULK1 complex not only provides a scaffold platform for AMPK and ULK1 but also enhances AMPK activity by recruiting SESN2, ultimately promoting autophagy activation.

Given that AMPK has multiple ligand-binding sites and can be regulated by activators at different sites, this provides an important molecular basis for developing therapeutic strategies based on multi-site synergistic activation. Studies have found that the combination of low-dose metformin and salicylate significantly activates AMPK and enhances ACC phosphorylation levels, effectively reducing hepatic triglyceride content and improving MASLD phenotype in high-fat diet-induced obese mouse models, while neither drug alone at the same dosage produced such therapeutic effects (121). Therefore, synergistic activation of the nucleotide-binding site and ADaM site may be one of the effective strategies for maximizing AMPK activation, though its efficacy and safety need further validation through clinical trials.

## 5 Summary and perspective

As a highly conserved metabolic stress sensor in evolution, AMPK has been extensively studied over the past decades, with continuous revelations of its structural characteristics, regulatory mechanisms, and downstream effects. This accumulated basic research has greatly enhanced our understanding of AMPK’s role in MASLD pathogenesis while establishing a solid foundation for developing AMPK-targeted therapies. Based on existing evidence, AMPK activation exhibits protective effects against MASLD through regulation of multiple pathways, including lipid metabolism, mitochondrial function, cellular autophagy, and inflammatory responses, demonstrating its potential as a therapeutic target. Unfortunately, no AMPK activators have yet been approved for clinical treatment of MASLD. This field still faces numerous challenges, such as: current ADaM site activators show insufficient affinity for the predominantly expressed AMPK complexes in the liver, potentially limiting therapeutic efficacy; non-selective activation of systemic AMPK may cause adverse effects, such as promoting cardiac hypertrophy (122); and considering MASLD’s heterogeneous characteristics, the suitable patient population for AMPK-targeted therapy needs further clarification.

Addressing these issues, several research directions and technological advances in recent years warrant attention, in addition to conducting larger-scale clinical trials. First, in-depth studies of physiological AMPK activation modes such as exercise and caloric restriction (CR), and the subsequent development of exercise mimetics and CR mimetics, may provide new insights for developing safer AMPK activation strategies (100, 103, 123). Second, the application of novel drug delivery technologies may improve the tissue selectivity of AMPK activators (124). For example, galactose-modified nanocarriers significantly enhance hepatic accumulation of the AMPK activator resveratrol, improving insulin sensitivity in MASLD mice. This strategy shows promise in overcoming both low bioavailability and off-target effects. Additionally, Ngoei et al. (125) reported a small molecule activator SC4 with high affinity for β2 subunit-containing complexes. Crystal structure analysis of α2β2γ1 bound to SC4 revealed that the 4′-nitrogen in SC4’s core mediates binding with β2 residue Asp111. Although SC4 did not significantly activate the predominantly expressed α1β2γ1 complex in hepatocytes in cell-free assays, the structural analysis of selectivity differences between SC4 and other ADaM site activators may provide new clues for developing specific activators targeting the α1β2γ1 complex.
